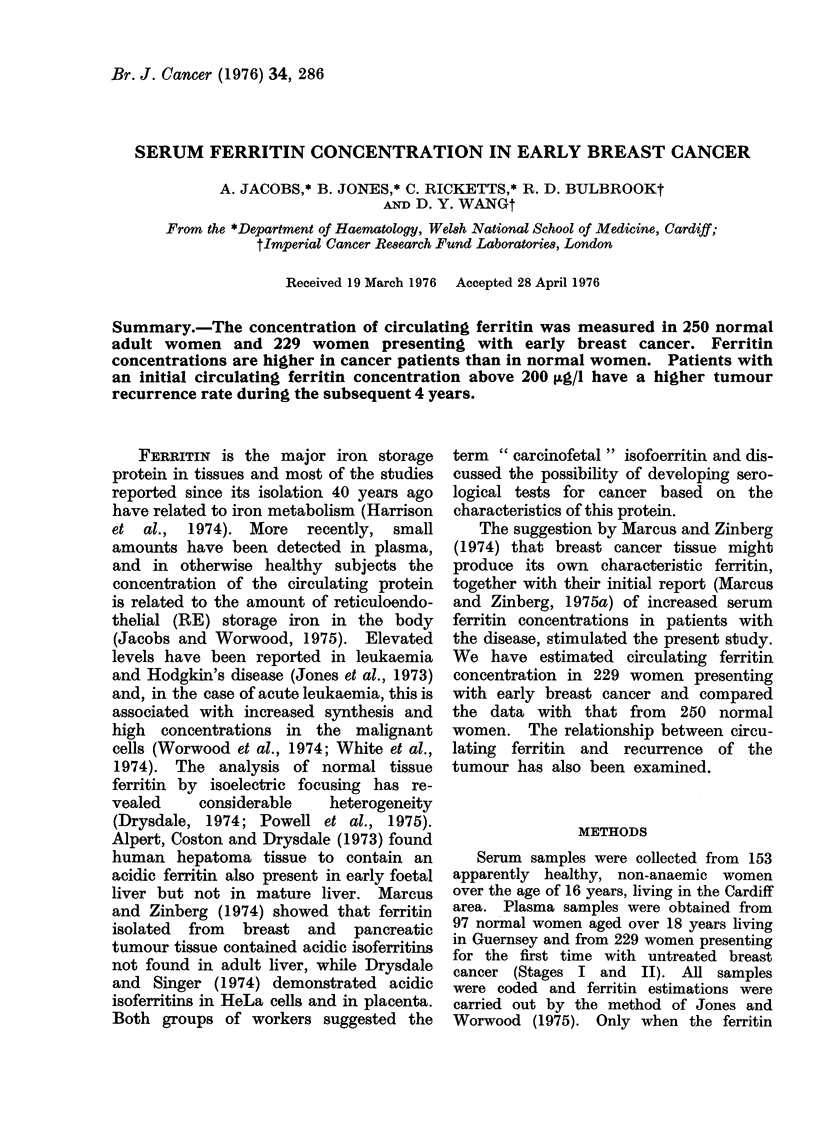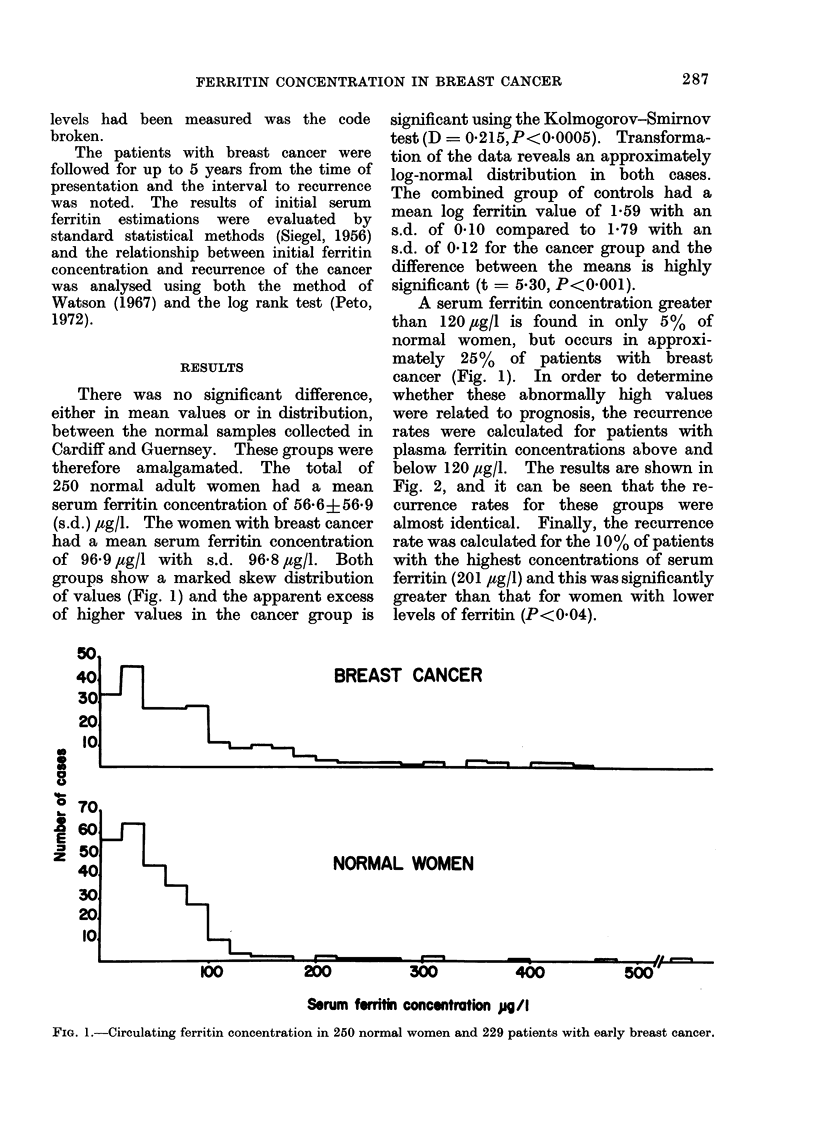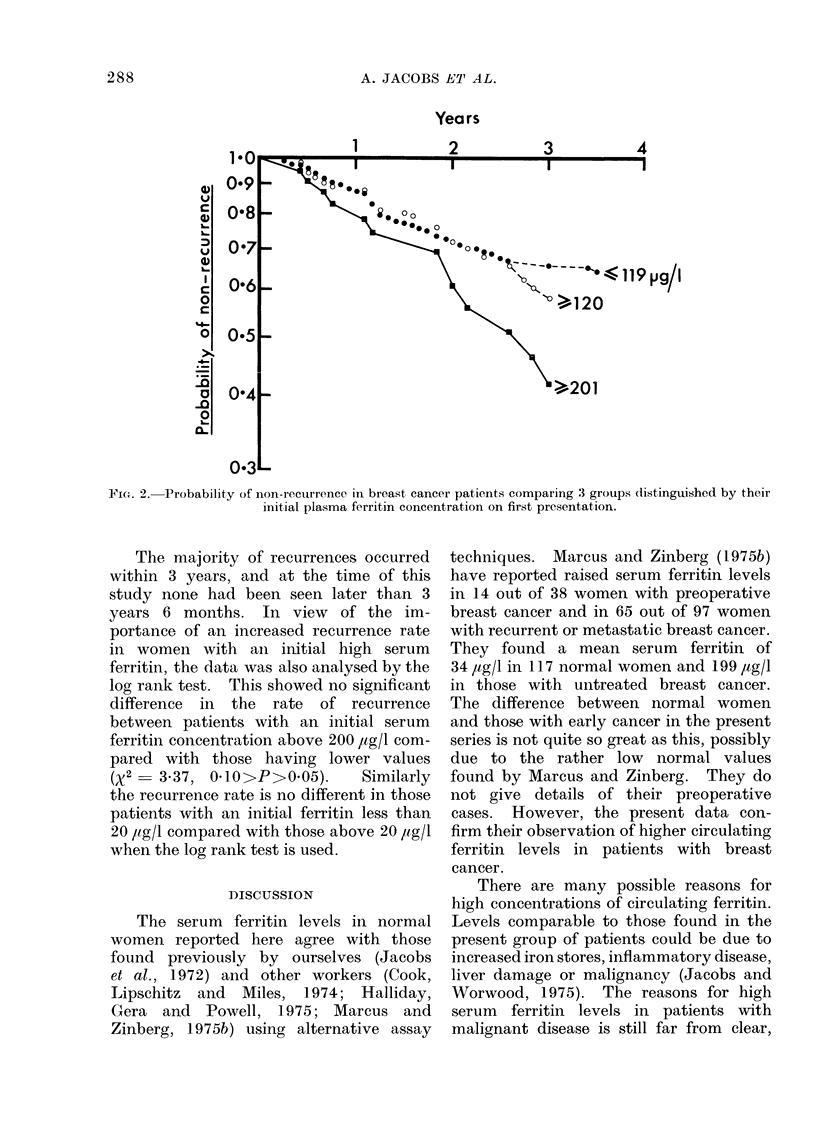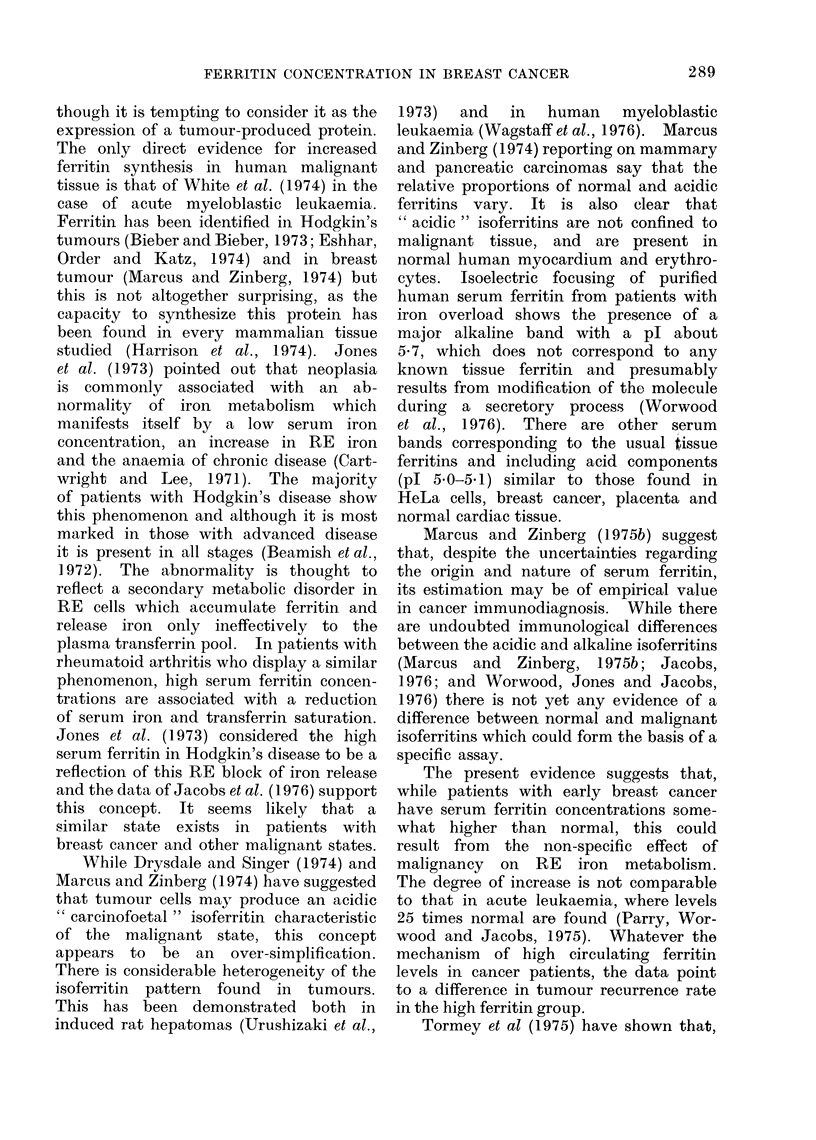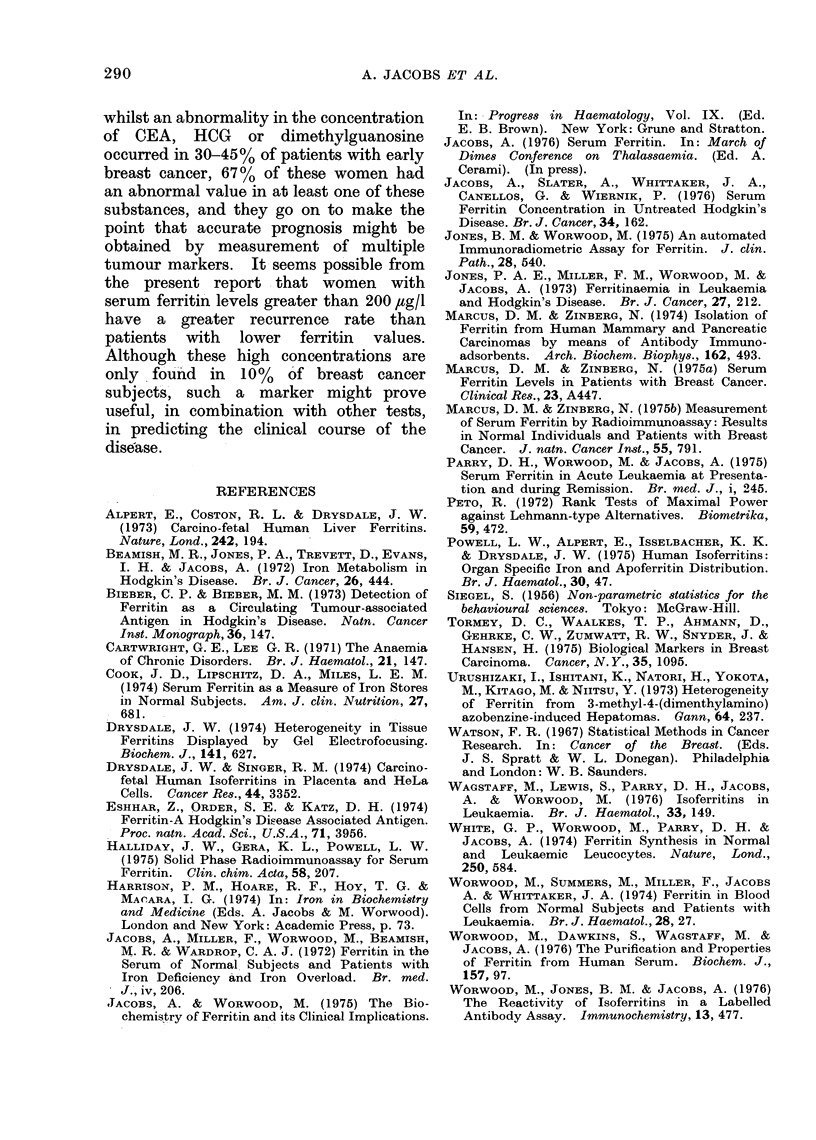# Serum ferritin concentration in early breast cancer.

**DOI:** 10.1038/bjc.1976.164

**Published:** 1976-09

**Authors:** A. Jacobs, B. Jones, C. Ricketts, R. D. Bulbrook, D. Y. Wang

## Abstract

The concentration of circulating ferritin was measured in 250 normal adult women and 229 women presenting with early breast cancer. Ferritin concentrations are higher in cancer patients than in normal women. Patients with an intial circulating ferritin concentration above 200 mug/1 have a higher tumour recurrence rate during the subsequent 4 years.


Br. J. Cancer (1976) 34, 286

### SERUM FERRITIN CONCENTRATION IN EARLY BREAST CANCER

A. JACOBS,* B. JONES,* C. RICKETTS,* R. D. BULBROOK† AND D. Y. WANG†

*From the *Department of Haematology, Welsh National School of Medicine, Cardiff; †Imperial Cancer Research Fund Laboratories, London*

Received 19 March 1976 Accepted 28 April 1976

**Summary.—The concentration of circulating ferritin was measured in 250 normal adult women and 229 women presenting with early breast cancer. Ferritin concentrations are higher in cancer patients than in normal women. Patients with an initial circulating ferritin concentration above 200 μg/l have a higher tumour recurrence rate during the subsequent 4 years.**

FERRITIN is the major iron storage protein in tissues and most of the studies reported since its isolation 40 years ago have related to iron metabolism (Harrison *et al.*, 1974). More recently, small amounts have been detected in plasma, and in otherwise healthy subjects the concentration of the circulating protein is related to the amount of reticuloendothelial (RE) storage iron in the body (Jacobs and Worwood, 1975). Elevated levels have been reported in leukaemia and Hodgkin's disease (Jones *et al.*, 1973) and, in the case of acute leukaemia, this is associated with increased synthesis and high concentrations in the malignant cells (Worwood *et al.*, 1974; White *et al.*, 1974). The analysis of normal tissue ferritin by isoelectric focusing has revealed considerable heterogeneity (Drysdale, 1974; Powell *et al.*, 1975). Alpert, Coston and Drysdale (1973) found human hepatoma tissue to contain an acidic ferritin also present in early foetal liver but not in mature liver. Marcus and Zinberg (1974) showed that ferritin isolated from breast and pancreatic tumour tissue contained acidic isoferritins not found in adult liver, while Drysdale and Singer (1974) demonstrated acidic isoferritins in HeLa cells and in placenta. Both groups of workers suggested the term "carcinofetal" isofoerritin and discussed the possibility of developing serological tests for cancer based on the characteristics of this protein.

The suggestion by Marcus and Zinberg (1974) that breast cancer tissue might produce its own characteristic ferritin, together with their initial report (Marcus and Zinberg, 1975*a*) of increased serum ferritin concentrations in patients with the disease, stimulated the present study. We have estimated circulating ferritin concentration in 229 women presenting with early breast cancer and compared the data with that from 250 normal women. The relationship between circulating ferritin and recurrence of the tumour has also been examined.

### METHODS

Serum samples were collected from 153 apparently healthy, non-anaemic women over the age of 16 years, living in the Cardiff area. Plasma samples were obtained from 97 normal women aged over 18 years living in Guernsey and from 229 women presenting for the first time with untreated breast cancer (Stages I and II). All samples were coded and ferritin estimations were carried out by the method of Jones and Worwood (1975). Only when the ferritin levels had been measured was the code broken.

The patients with breast cancer were followed for up to 5 years from the time of presentation and the interval to recurrence was noted. The results of initial serum ferritin estimations were evaluated by standard statistical methods (Siegel, 1956) and the relationship between initial ferritin concentration and recurrence of the cancer was analysed using both the method of Watson (1967) and the log rank test (Peto, 1972).

### RESULTS

There was no significant difference, either in mean values or in distribution, between the normal samples collected in Cardiff and Guernsey. These groups were therefore amalgamated. The total of 250 normal adult women had a mean serum ferritin concentration of $56\cdot6 \pm 56\cdot9$ (s.d.) $\mu$g/l. The women with breast cancer had a mean serum ferritin concentration of $96\cdot9$ $\mu$g/l with s.d. $96\cdot8$ $\mu$g/l. Both groups show a marked skew distribution of values (Fig. 1) and the apparent excess of higher values in the cancer group is significant using the Kolmogorov–Smirnov test ($D = 0\cdot215, P < 0\cdot0005$). Transformation of the data reveals an approximately log-normal distribution in both cases. The combined group of controls had a mean log ferritin value of 1·59 with an s.d. of 0·10 compared to 1·79 with an s.d. of 0·12 for the cancer group and the difference between the means is highly significant ($t = 5\cdot30, P < 0\cdot001$).

A serum ferritin concentration greater than 120 $\mu$g/l is found in only 5% of normal women, but occurs in approximately 25% of patients with breast cancer (Fig. 1). In order to determine whether these abnormally high values were related to prognosis, the recurrence rates were calculated for patients with plasma ferritin concentrations above and below 120 $\mu$g/l. The results are shown in Fig. 2, and it can be seen that the recurrence rates for these groups were almost identical. Finally, the recurrence rate was calculated for the 10% of patients with the highest concentrations of serum ferritin (201 $\mu$g/l) and this was significantly greater than that for women with lower levels of ferritin ($P < 0\cdot04$).

Fig. 1.—Circulating ferritin concentration in 250 normal women and 229 patients with early breast cancer.

FIG. 2.—Probability of non-recurrence in breast cancer patients comparing 3 groups distinguished by their initial plasma ferritin concentration on first presentation.

The majority of recurrences occurred within 3 years, and at the time of this study none had been seen later than 3 years 6 months. In view of the importance of an increased recurrence rate in women with an initial high serum ferritin, the data was also analysed by the log rank test. This showed no significant difference in the rate of recurrence between patients with an initial serum ferritin concentration above 200 μg/l compared with those having lower values ($\chi^2 = 3{\cdot}37$, $0{\cdot}10 > P > 0{\cdot}05$). Similarly the recurrence rate is no different in those patients with an initial ferritin less than 20 μg/l compared with those above 20 μg/l when the log rank test is used.

### DISCUSSION

The serum ferritin levels in normal women reported here agree with those found previously by ourselves (Jacobs *et al.*, 1972) and other workers (Cook, Lipschitz and Miles, 1974; Halliday, Gera and Powell, 1975; Marcus and Zinberg, 1975*b*) using alternative assay techniques. Marcus and Zinberg (1975*b*) have reported raised serum ferritin levels in 14 out of 38 women with preoperative breast cancer and in 65 out of 97 women with recurrent or metastatic breast cancer. They found a mean serum ferritin of 34 μg/l in 117 normal women and 199 μg/l in those with untreated breast cancer. The difference between normal women and those with early cancer in the present series is not quite so great as this, possibly due to the rather low normal values found by Marcus and Zinberg. They do not give details of their preoperative cases. However, the present data confirm their observation of higher circulating ferritin levels in patients with breast cancer.

There are many possible reasons for high concentrations of circulating ferritin. Levels comparable to those found in the present group of patients could be due to increased iron stores, inflammatory disease, liver damage or malignancy (Jacobs and Worwood, 1975). The reasons for high serum ferritin levels in patients with malignant disease is still far from clear, though it is tempting to consider it as the expression of a tumour-produced protein. The only direct evidence for increased ferritin synthesis in human malignant tissue is that of White *et al.* (1974) in the case of acute myeloblastic leukaemia. Ferritin has been identified in Hodgkin's tumours (Bieber and Bieber, 1973; Eshhar, Order and Katz, 1974) and in breast tumour (Marcus and Zinberg, 1974) but this is not altogether surprising, as the capacity to synthesize this protein has been found in every mammalian tissue studied (Harrison *et al.*, 1974). Jones *et al.* (1973) pointed out that neoplasia is commonly associated with an abnormality of iron metabolism which manifests itself by a low serum iron concentration, an increase in RE iron and the anaemia of chronic disease (Cartwright and Lee, 1971). The majority of patients with Hodgkin's disease show this phenomenon and although it is most marked in those with advanced disease it is present in all stages (Beamish *et al.*, 1972). The abnormality is thought to reflect a secondary metabolic disorder in RE cells which accumulate ferritin and release iron only ineffectively to the plasma transferrin pool. In patients with rheumatoid arthritis who display a similar phenomenon, high serum ferritin concentrations are associated with a reduction of serum iron and transferrin saturation. Jones *et al.* (1973) considered the high serum ferritin in Hodgkin's disease to be a reflection of this RE block of iron release and the data of Jacobs *et al.* (1976) support this concept. It seems likely that a similar state exists in patients with breast cancer and other malignant states.

While Drysdale and Singer (1974) and Marcus and Zinberg (1974) have suggested that tumour cells may produce an acidic " carcinofoetal " isoferritin characteristic of the malignant state, this concept appears to be an over-simplification. There is considerable heterogeneity of the isoferritin pattern found in tumours. This has been demonstrated both in induced rat hepatomas (Urushizaki *et al.*, 1973) and in human myeloblastic leukaemia (Wagstaff *et al.*, 1976). Marcus and Zinberg (1974) reporting on mammary and pancreatic carcinomas say that the relative proportions of normal and acidic ferritins vary. It is also clear that " acidic " isoferritins are not confined to malignant tissue, and are present in normal human myocardium and erythrocytes. Isoelectric focusing of purified human serum ferritin from patients with iron overload shows the presence of a major alkaline band with a pI about 5·7, which does not correspond to any known tissue ferritin and presumably results from modification of the molecule during a secretory process (Worwood *et al.*, 1976). There are other serum bands corresponding to the usual tissue ferritins and including acid components (pI 5·0–5·1) similar to those found in HeLa cells, breast cancer, placenta and normal cardiac tissue.

Marcus and Zinberg (1975*b*) suggest that, despite the uncertainties regarding the origin and nature of serum ferritin, its estimation may be of empirical value in cancer immunodiagnosis. While there are undoubted immunological differences between the acidic and alkaline isoferritins (Marcus and Zinberg, 1975*b*; Jacobs, 1976; and Worwood, Jones and Jacobs, 1976) there is not yet any evidence of a difference between normal and malignant isoferritins which could form the basis of a specific assay.

The present evidence suggests that, while patients with early breast cancer have serum ferritin concentrations somewhat higher than normal, this could result from the non-specific effect of malignancy on RE iron metabolism. The degree of increase is not comparable to that in acute leukaemia, where levels 25 times normal are found (Parry, Worwood and Jacobs, 1975). Whatever the mechanism of high circulating ferritin levels in cancer patients, the data point to a difference in tumour recurrence rate in the high ferritin group.

Tormey *et al* (1975) have shown that, whilst an abnormality in the concentration of CEA, HCG or dimethylguanosine occurred in 30–45% of patients with early breast cancer, 67% of these women had an abnormal value in at least one of these substances, and they go on to make the point that accurate prognosis might be obtained by measurement of multiple tumour markers. It seems possible from the present report that women with serum ferritin levels greater than 200 $\mu$g/l have a greater recurrence rate than patients with lower ferritin values. Although these high concentrations are only found in 10% of breast cancer subjects, such a marker might prove useful, in combination with other tests, in predicting the clinical course of the disease.

### REFERENCES

ALPERT, E., COSTON, R. L. & DRYSDALE, J. W. (1973) Carcino-fetal Human Liver Ferritins. *Nature, Lond.*, **242**, 194.

BEAMISH, M. R., JONES, P. A., TREVETT, D., EVANS, I. H. & JACOBS, A. (1972) Iron Metabolism in Hodgkin's Disease. *Br. J. Cancer*, **26**, 444.

BIEBER, C. P. & BIEBER, M. M. (1973) Detection of Ferritin as a Circulating Tumour-associated Antigen in Hodgkin's Disease. *Natn. Cancer Inst. Monograph*, **36**, 147.

CARTWRIGHT, G. E., LEE G. R. (1971) The Anaemia of Chronic Disorders. *Br. J. Haematol.*, **21**, 147.

COOK, J. D., LIPSCHITZ, D. A., MILES, L. E. M. (1974) Serum Ferritin as a Measure of Iron Stores in Normal Subjects. *Am. J. clin. Nutrition*, **27**, 681.

DRYSDALE, J. W. (1974) Heterogeneity in Tissue Ferritins Displayed by Gel Electrofocusing. *Biochem. J.*, **141**, 627.

DRYSDALE, J. W. & SINGER, R. M. (1974) Carcino-fetal Human Isoferritins in Placenta and HeLa Cells. *Cancer Res.*, **44**, 3352.

ESHHAR, Z., ORDER, S. E. & KATZ, D. H. (1974) Ferritin-A Hodgkin's Disease Associated Antigen. *Proc. natn. Acad. Sci., U.S.A.*, **71**, 3956.

HALLIDAY, J. W., GERA, K. L., POWELL, L. W. (1975) Solid Phase Radioimmunoassay for Serum Ferritin. *Clin. chim. Acta*, **58**, 207.

HARRISON, P. M., HOARE, R. F., HOY, T. G. & MACARA, I. G. (1974) In: *Iron in Biochemistry and Medicine* (Eds. A. Jacobs & M. Worwood). London and New York: Academic Press, p. 73.

JACOBS, A., MILLER, F., WORWOOD, M., BEAMISH, M. R. & WARDROP, C. A. J. (1972) Ferritin in the Serum of Normal Subjects and Patients with Iron Deficiency and Iron Overload. *Br. med. J.*, iv, 206.

JACOBS, A. & WORWOOD, M. (1975) The Biochemistry of Ferritin and its Clinical Implications. In: *Progress in Haematology*, Vol. IX. (Ed. E. B. Brown). New York: Grune and Stratton.

JACOBS, A. (1976) Serum Ferritin. In: *March of Dimes Conference on Thalassaemia.* (Ed. A. Cerami). (In press).

JACOBS, A., SLATER, A., WHITTAKER, J. A., CANELLOS, G. & WIERNIK, P. (1976) Serum Ferritin Concentration in Untreated Hodgkin's Disease. *Br. J. Cancer*, **34**, 162.

JONES, B. M. & WORWOOD, M. (1975) An automated Immunoradiometric Assay for Ferritin. *J. clin. Path.*, **28**, 540.

JONES, P. A. E., MILLER, F. M., WORWOOD, M. & JACOBS, A. (1973) Ferritinaemia in Leukaemia and Hodgkin's Disease. *Br. J. Cancer*, **27**, 212.

MARCUS, D. M. & ZINBERG, N. (1974) Isolation of Ferritin from Human Mammary and Pancreatic Carcinomas by means of Antibody Immuno-adsorbents. *Arch. Biochem. Biophys.*, **162**, 493.

MARCUS, D. M. & ZINBERG, N. (1975*a*) Serum Ferritin Levels in Patients with Breast Cancer. *Clinical Res.*, **23**, A447.

MARCUS, D. M. & ZINBERG, N. (1975*b*) Measurement of Serum Ferritin by Radioimmunoassay: Results in Normal Individuals and Patients with Breast Cancer. *J. natn. Cancer Inst.*, **55**, 791.

PARRY, D. H., WORWOOD, M. & JACOBS, A. (1975) Serum Ferritin in Acute Leukaemia at Presentation and during Remission. *Br. med. J.*, i, 245.

PETO, R. (1972) Rank Tests of Maximal Power against Lehmann-type Alternatives. *Biometrika*, **59**, 472.

POWELL, L. W., ALPERT, E., ISSELBACHER, K. K. & DRYSDALE, J. W. (1975) Human Isoferritins: Organ Specific Iron and Apoferritin Distribution. *Br. J. Haematol.*, **30**, 47.

SIEGEL, S. (1956) *Non-parametric statistics for the behavioural sciences.* Tokyo: McGraw-Hill.

TORMEY, D. C., WAALKES, T. P., AHMANN, D., GEHRKE, C. W., ZUMWATT, R. W., SNYDER, J. & HANSEN, H. (1975) Biological Markers in Breast Carcinoma. *Cancer, N.Y.*, **35**, 1095.

URUSHIZAKI, I., ISHITANI, K., NATORI, H., YOKOTA, M., KITAGO, M. & NIITSU, Y. (1973) Heterogeneity of Ferritin from 3-methyl-4-(dimenthylamino) azobenzine-induced Hepatomas. *Gann*, **64**, 237.

WATSON, F. R. (1967) Statistical Methods in Cancer Research. In: *Cancer of the Breast.* (Eds. J. S. Spratt & W. L. Donegan). Philadelphia and London: W. B. Saunders.

WAGSTAFF, M., LEWIS, S., PARRY, D. H., JACOBS, A. & WORWOOD, M. (1976) Isoferritins in Leukaemia. *Br. J. Haematol.*, **33**, 149.

WHITE, G. P., WORWOOD, M., PARRY, D. H. & JACOBS, A. (1974) Ferritin Synthesis in Normal and Leukaemic Leucocytes. *Nature, Lond.*, **250**, 584.

WORWOOD, M., SUMMERS, M., MILLER, F., JACOBS A. & WHITTAKER, J. A. (1974) Ferritin in Blood Cells from Normal Subjects and Patients with Leukaemia. *Br. J. Haematol.*, **28**, 27.

WORWOOD, M., DAWKINS, S., WAGSTAFF, M. & JACOBS, A. (1976) The Purification and Properties of Ferritin from Human Serum. *Biochem. J.*, **157**, 97.

WORWOOD, M., JONES, B. M. & JACOBS, A. (1976) The Reactivity of Isoferritins in a Labelled Antibody Assay. *Immunochemistry*, **13**, 477.